# Non-Suicidal Self-Injury Among Incarcerated Adolescents: Prevalence, Personality, and Psychiatric Comorbidity

**DOI:** 10.3389/fpsyt.2021.652004

**Published:** 2021-05-19

**Authors:** Roman Koposov, Andrew Stickley, Vladislav Ruchkin

**Affiliations:** ^1^Regional Centre for Child and Youth Mental Health and Child Welfare, Faculty of Health Sciences, UiT The Arctic University of Norway, Tromsø, Norway; ^2^Department of Epidemiology and Modern Technologies of Vaccination, Institute of Professional Education, Sechenov First Moscow State Medical University, Moscow, Russia; ^3^Department of Preventive Intervention for Psychiatric Disorders, National Center of Neurology and Psychiatry, Kodaira, Japan; ^4^Stockholm Center for Health and Social Change (SCOHOST), Södertörn University, Huddinge, Sweden; ^5^Child and Adolescent Psychiatry Unit, Department of Neuroscience, Uppsala University, Uppsala, Sweden; ^6^Child Study Center, Yale School of Medicine, New Haven, CT, United States; ^7^Säter Forensic Psychiatric Clinic, Säter, Sweden

**Keywords:** non-suicidal self-injury, psychopathology, personality, incarcerated adolescents, Russia

## Abstract

**Introduction:** Incarcerated adolescents represent a risk group for non-suicidal self-injury (NSSI), but research on this population has been limited and no studies have been conducted in Russia. To address this deficit, this study examined NSSI and the factors associated with it among youth in a juvenile correctional facility in Russia.

**Methods:** NSSI and psychopathology were assessed using a psychiatric interview and self-report questionnaire in 368 incarcerated male adolescents aged 14–19 years (mean age 16.4 years, S.D. 0.9) from Northern Russia.

**Results:** 18.2% (*N* = 67) of the study participants had a history of NSSI and also had higher rates of anxiety, post-traumatic stress disorder (PTSD), depression, community violence exposure and scored higher on most of the Youth Self-Report problem scales. In addition, 31.3% of the NSSI group reported previous suicidal ideation and had thought about a specific suicide method compared to 12.0% in the No-NSSI group. Adolescents with NSSI also differed significantly from the No-NSSI group on self-directedness (lower) and self-transcendence (higher) personality traits.

**Conclusion:** NSSI is common in incarcerated adolescents in Russia and is associated with extensive psychiatric comorbidity, suicidal ideation and specific personality traits.

## Introduction

Non-suicidal self-injury (NSSI), which involves deliberate, self-inflicted damage of body tissue without suicidal intent, is a serious and prevalent problem (1). Research has shown that NSSI most commonly occurs among 13–15-year olds (2–4), while other studies have highlighted the severe psychological, medical, social, legal and ethical consequences associated with NSSI (5–8). Several international studies have examined NSSI in adolescent populations in different samples. Lifetime prevalence rates of NSSI of 17–18% in adolescents were reported in two systematic reviews (9, 10), with considerable international variability across community samples [from 2.9 to 42% in (9) and from 9.4 to 49.2 in (10)]. In clinical samples the numbers tend to be higher with the prevalence rates varying between 50 and 61% (11, 12). Another review (13) suggested that the aggregate lifetime prevalence of NSSI tended to be higher in non-Western (32.6%) than Western countries (19.4%). Despite this research, there are still many settings where there has been little focus on NSSI. This is the case for the Russian Federation where few studies have been conducted so far and national statistics on the prevalence of NSSI are limited (14, 15). Nevertheless, there is some indication that NSSI might be prevalent in Russia as a recent study found that 13% of adolescents reported at least one previous episode of self-injury (16).

Associations between self-injury and mental health problems are common and the vast majority of adolescents who engage in NSSI also have some degree of comorbidity, including post-traumatic stress disorder (PTSD) (17, 18), with generally higher levels of traumatic exposure (19, 20), depressive disorders (21), obsessive-compulsive disorder (18), anxiety (22), symptoms of borderline personality disorder (23, 24), specific phobia (21), autism (25), intellectual disability (26), schizophrenia (27), and eating disorders (28). A strong association has also been noted between NSSI and externalizing problems where conduct disorder (CD) and attention deficit hyperactivity disorder (ADHD) are most common (29, 30). Adolescents engaging in NSSI may also have substantial difficulties in social interaction, such as problems with peers and poor academic achievement at school (31, 32). These conditions may be related to worse NSSI in terms of frequency/severity and all represent detrimental outcomes that can complicate efforts to treat NSSI or exacerbate the effects of NSSI.

An additional problem is posed by the possible association between self-injurious behavior and suicide. While some research has suggested that a large proportion of self-injury cases tend to be non-suicidal in nature and that self-injurious behavior is intended to effect a change in the environment, rather than result in an individual's death (33), other studies have consistently reported that NSSI is a robust predictor of suicidality (34–37) with suicidal ideation and attempts being more common among individuals with NSSI as compared to individuals without NSSI (2, 38). It has also been found that NSSI predicts suicidal behavior over and above mental health problems, such as depression (35), hopelessness and borderline personality traits (34), PTSD and a history of child abuse (39).

Among the most well-known and widely researched factors linked to self-harming behavior are emotion regulation (40) and impulsivity (41), where NSSI is thought to occur as a result of emotional and cognitive dysregulation (42), as well as impaired impulse control (43). From this perspective, personality has been thought to influence the use of emotion regulation strategies (44) and the ability to delay actions in order to plan and make choices by identifying a solution (43). Indeed, personality research among the general population using Cloninger's biopsychosocial personality model (45) has demonstrated a relationship between personality traits and emotion regulation as well as impulsive behavior (46–48). Regarding NSSI, it has been reported that specific personality traits are associated with NSSI in adolescents (49, 50). Tschan et al. (51) found that adolescents with a history of NSSI had a personality profile that included higher scores on the personality traits novelty seeking (NS) and harm avoidance (HA) and lower scores on the character traits self-directedness (SD), persistence (P) and cooperativeness (C) compared to adolescents with mental disorders but without NSSI. In addition, another study (52) found that while adolescents with occasional and repetitive NSSI differed significantly from those without NSSI only on low SD, individuals with repetitive NSSI differed on several personality traits, such as higher NS, HA and self-transcendence (ST) and lower SD. These findings suggest that researchers and clinicians might benefit from identifying personality traits that are linked to NSSI in order to gain a better understanding of the mechanisms that are involved in NSSI and to develop treatment approaches that consider personality (53, 54). Self-injurious behavior is a serious problem in offender populations (55). Nonetheless, while reviews on NSSI among incarcerated adults are available (56, 57), the comparable literature for adolescents is still somewhat limited. Several studies have suggested that youth from the juvenile justice system may represent an especially high risk population for self-injurious and suicidal behavior (17, 58, 59). Two studies (60, 61), included in a review by Casiano et al. (62), reported a lifetime prevalence of “deliberate self-harm (acts of both suicidal and non-suicidal self-injury)” in incarcerated juveniles that ranged from 6 to 40%. A high prevalence of self-harm among young offenders is not surprising given that many of the risk factors for NSSI, such as poverty, violence and poor mental health, exist at higher rates among this population when compared to the general youth population (63). Moore et al. (64) suggested that cumulative exposure to multiple risk factors, which may take place among youth in custody, might further increase the risk of NSSI.

A number of studies have explored factors associated with suicidal ideation and attempts among delinquent youths (65, 66), but as yet, research on NSSI has been limited, and no previous study has investigated the prevalence of NSSI among incarcerated youth in Russia. Expanded knowledge regarding different aspects of NSSI among incarcerated youth, such as its prevalence, comorbidity and association with personality, can potentially facilitate the development of more effective interventions for self-harming behavior.

The current study aimed to: (1) estimate the prevalence of NSSI in incarcerated adolescents in Russia; (2) evaluate what types of psychiatric diagnoses and self-reported mental health problems are associated with a history of NSSI; and (3) examine whether specific personality traits are related to NSSI in this population. We expected that adolescents from the NSSI group would have higher rates of comorbid psychopathology and suicidal ideation. We also expected that NSSI would be associated with specific personality traits.

## Materials and Methods

### Participants

Study participants were recruited during a half-year period from male adolescent inmates, who after a court decision, had been sent to a juvenile correctional facility in the Arkhangelsk region (located in Northern Russia, with a population of 1.5 million people, of whom, 98% are ethnic Slavs). In Russia, full criminal responsibility starts at 16 years of age (but at 14 years of age for the most dangerous crimes), and in case of longer sentences juveniles can be transferred to an adult facility after they become 18, but are often allowed to remain in the juvenile facility until they are 19 years old. Property crimes (51%), violence-related crimes (38%), and rape/sexual violence (6%) or murder (5%) were among the most common offenses in those individuals who participated in this study. Generally, those sentenced for theft had shown a repetitive pattern of stealing and had multiple convictions with sentencing to the facility usually taking place after a repeat conviction while on parole. The mean sentence length at the time of the study was 4.3 years, while all participants had been institutionalized (including the detention period) for at least 6 months. The age of the participants (*N* = 368) ranged from 14 to 19 years (mean age 16.4, S.D. 0.9).

As in all Russian juvenile correctional facilities, the incarcerated youth were divided into companies of 100, each of which was further divided into sub-groups of 25 youths. Every company occupies one floor of a two-story housing building. Inside each building where the youths eat, sleep and have their lessons, there are also recreation and training rooms. Several other units are located on the territory of the correctional facility including a school, medical and kitchen units, library, bath unit, etc. Youth are under constant supervision and cannot move around the facility freely.

The research was approved by the ethical committee from the Northern State Medical University in Arkhangelsk (Russia). Participants were assured about the voluntary and confidential nature of their participation and that no personal information or information relating to their responses would be given to the staff at the correctional facility. Detailed information about the study was provided to the participants before their consent was obtained. Eight inmates refused to participate because of an unwillingness to provide personal information. Oral informed consent was obtained from all the participants and in addition, also from the director of the juvenile correctional facility.

A semi-structured psychiatric interview (K-SADS-PL) was conducted to assess mental health problems among the 368 delinquent youths who participated in the study. While the psychiatric assessment was conducted individually, questionnaires were filled out in small group sessions (5–8 participants). During the interview and while filling out self-reports, the adolescents did not express any difficulties or worries related to the content of the questions nor did they express any visible discomfort while being interviewed about their mental health. Personal and direct communication with each given adolescent helped us to establish trusting and cooperative relationships. Individuals with current suicidal ideation were offered psychological/psychiatric help at the facility. Anybody who was judged as a clear and immediate suicide risk but refused help would have been reported to the psychiatrist at the facility, irrespective of their wishes, as the situation would have been considered life-threatening. Fortunately, no cases were identified that required an immediate intervention, and only a few youths asked for a referral to a mental health professional. No suicide attempts or acts of self-harm occurred in the facility during the study period.

### Instruments

#### Lifetime NSSI

The depressive disorders part of the Schedule for Affective Disorders and Schizophrenia for School-Age Children-Present and Lifetime Version (K-SADS-PL) (67) contains five items inquiring about self-injury and suicide, among which, one item assesses non-suicidal physical self-damaging acts [described in all future analyses as non-suicidal self-injury (NSSI)]. These refer to self-mutilation, or self-harmful behavior where there is no intent to die, while four items enquire about suicidal ideation and attempts (“Thoughts of death and/or wish to die”; “Thinking about killing oneself with or without a specific plan”; “Attempts to kill oneself and wish to die during the attempt”; “Seriousness and severity of the attempt”).

The *No-NSSI group* consisted of those participants who answered negatively to the question about hurting themselves (and were coded 0). Those who described infrequent (1–3 times a year) self-injurious episodes and had never caused serious injury to themselves, and those who reported frequent (4 or more times a year) self-injury episodes or had caused serious injury to themselves, were combined into the *NSSI group* (coded as 1).

#### Psychopathology

Current and past psychiatric diagnoses were obtained with the K-SADS-PL (67). For a diagnosis to be made, diagnosis-specific impairment that was clinically significant had to be present (68, 69). Two psychiatrists conducted the interviews using DSM-IV criteria (70). Previous studies have shown that interrater reliability is high for this instrument, with 94–100% interrater agreement in scoring screens and diagnoses (67). As this study aimed to assess the association between lifetime NSSI and psychopathology, lifetime rates of psychiatric diagnoses were used in the analysis.

#### Witnessing and Victimization

A modified version of the Survey of Exposure to Community Violence (71) was used to assess different types of violence exposure (e.g., “Seen somebody/was beaten up or mugged,” “Threatened with serious physical harm,” etc.). The response alternatives reflect the number of episodes of exposure and range from 1 (never) to 9 (almost every day). As the scale represents an index rather than a rating scale (where being exposed to one type of violence does not necessarily suggest exposure to any other type), the scale's internal consistency was not evaluated.

#### Behavioral/Emotional Problems

The Youth Self-Report [YSR, (72)], which includes 122 items, was used to assess internalizing (withdrawn, somatic complaints, anxious/depressed scales) and externalizing (delinquent and aggressive behavior) problems. Items are scored on a 3-point scale, ranging from 0 (not true) to 2 (very true or often true) with higher scores indicating greater problems.

#### Depressive Symptoms

The Beck Depression Inventory [BDI, (73)] consists of 20 items with four response alternatives (ranging from 0 to 3) describing individual feelings and behaviors related to depression, with higher scores indicating increased depressive symptoms. Earlier research has shown that the BDI has acceptable psychometric properties in both psychiatric and non-psychiatric samples (73). Cronbach's alpha for the scale in the present sample was 0.89.

*The UCLA Post-Traumatic Stress Disorder Reaction Index for Children and Adolescents (PTSD-RI)* is a self-report instrument designed to assess experiences and symptoms among children and adolescents exposed to traumatic events. The measure inquires about the frequency with which 20 specific post-traumatic stress symptoms occurred during the past month. This instrument, which is one of the most commonly utilized measures of childhood PTSD, has been widely used across countries (74). Cronbach's alpha for the scale was 0.87.

Personality traits were assessed with the *Temperament and Character Inventory* [TCI, (45, 75)], based on Cloninger's unified biosocial theory of personality (76). TCI measures four temperament dimensions—novelty seeking (NS), harm avoidance (HA), reward dependence (RD), and persistence (P); and three character dimensions—self-directedness (SD), cooperativeness (C), and self-transcendence (ST). According to this theory, while temperament traits are largely genetically determined, character traits depend on socialization processes and can change across the life course (45). The short 125 item version of the TCI with true or false answer options was used in this study. Cronbach's alphas for the various dimensions were—NS: 0.61, HA: 0.67, RD: 0.59, SD: 0.68, C: 0.64, ST: 0.75, P: 0.57. Due to low alpha values, RD and P (below 0.6) were not included in the present analysis.

## Translation

These scales were translated into Russian following established standard procedures (77). The Russian translation of the K-SADS-PL was done at the Department of Psychology, Moscow State University.

## Data Analysis

The data were analyzed using the Statistical Package for the Social Sciences (SPSS-25.0). Chi-square tests were used to compare the prevalence of psychiatric disorders between the NSSI and No-NSSI groups. Independent samples *t*-tests were used in order to compare the levels of problem scores between the groups. The level of statistical significance was *p* < 0.05 (two-tailed).

## Results

### Prevalence of NSSI and Suicide Ideation and Attempts

Of the 368 participating subjects, 67 (18.2%) described a history of NSSI. Of these, 23 (6.2%) described infrequent (1–3 times a year) self-injurious episodes and had never caused serious injury to themselves, and 44 (12%) reported frequent (4 or more times a year) self-injurious episodes or had caused serious harm to themselves. Since no significant differences were found between these two groups in terms of comorbid psychopathology, they were combined into the *NSSI group* (*N* = 67, coded as 1) in all further analyses.

When comparing the NSSI group with all other delinquent youths regarding suicidal ideation and attempts, 38.8% in the NSSI group reported having had recurrent thoughts of death, as compared to 15.0% in the No-NSSI group (Chi-square = 20.21; *p* < 0.001); 31.3% in the NSSI group reported frequent suicidal ideation and had thought of a specific method for suicide, as compared to 12.0% in the No-NSSI group (Chi-square = 16.91; *p* < 0.001); and 28.4% in the NSSI group reported definite suicidal intent when previously attempting suicide, as compared to 8.0% in the No-NSSI group (Chi-square = 30.94; *p* <0.001). In addition, 59.6% of those in the NSSI group, as compared to 42.0% in the No-NSSI group, considered themselves as having some form of psychiatric problem (Chi-square = 5.34; *p* < 0.05), 52.8% of the NSSI group reported having had psychiatric treatment in the past, as compared to 37.2% in the No-NSSI group (Chi-square = 4.43; *p* < 0.05), and 15.1% of the NSSI group reported having relatives who had a psychiatric contact in the past, compared to 5.7% in the No-NSSI group (Chi-square = 5.61; *p* < 0.05).

### Psychopathology

The prevalence of comorbid psychiatric diagnoses among incarcerated adolescents in the No-NSSI and NSSI groups is presented in Table 1. The NSSI group had higher rates of anxiety disorder and PTSD. The difference in the prevalence of major depressive disorder (MDD) was near significance level (*p* = 0.064), with a higher prevalence of MDD in the NSSI group. When the number of comorbid diagnoses was tallied for each subject and compared between the two groups there was a trend toward having three or more psychiatric diagnoses in the NSSI group as compared to the No-NSSI group (*p* = 0.055).

**Table 1:** Prevalence of lifetime psychiatric diagnoses in delinquent youth with and without NSSI^a^.

**Other diagnosis**	**NSSI (*N =* 67)**	**No-NSSI (*N =* 301)**	**Chi-square^**b**^ (p)^**c**^**
MDD^d^	12 (17.9%)	30 (10%)	3.42 (0.064)
Mania	8 (11.9%)	31 (10.3%)	0.16 (0.693)
Anxiety disorder	20 (29.9%)	35 (11.6%)	14.32 (0.000)
PTSD^e^	23 (34.3%)	63 (20.9%)	5.49 (0.019)
CD^f^	53 (79.1%)	216 (71.8%)	1.50 (0.220)
Early onset CD	20 (29.9%)	63 (20.9%)	2.50 (0.114)
ADHD^g^	10 (14.9%)	53 (17.6%)	0.28 (0.598)
Alcohol dependence	32 (47.8%)	118 (39.2%)	1.66 (0.197)
Other substance dependence	19 (28.4%)	59 (19.6%)	2.52 (0.113)
**Any psychiatric diagnosis:**			
1–2	32 (47.8%)	159 (52.8%)	5.79 (0.055)
3 or more	32 (47.8%)	105 (34.9%)	

a *NSSI, Non-suicidal self-injury*.

b *Chi-square, The chi-square test*.

c *p, Significance value*.

d *MDD, Major depressive disorder*.

e *PTSD, Post-traumatic stress disorder*.

f *CD, Conduct disorder*.

g *ADHD, Attention deficit hyperactivity disorder*.

### Problem Scores and Personality Traits in the NSSI and No-NSSI Groups

The mean scores for different mental health problems, as well as for personality traits among incarcerated adolescents from the No-NSSI and NSSI groups are presented in Table 2. The NSSI group scored higher on post-traumatic stress, depression and on all types of problems assessed by the YSR, except for withdrawn symptoms and delinquent behavior. The NSSI group reported that they more often witnessed and were a victim of violence. Delinquents with NSSI also scored significantly lower on self-directedness and higher on self-transcendence compared to subjects from the No-NSSI group.

**Table 2:** Results of *t*-tests comparing problem scores and personality characteristics in delinquents with and without NSSI^a^.

**Variables**	**NSSI M (SD)^**c**^**	**No-NSSI^**b**^ M (SD)**	***t*-test^**d**^ (df)^**e**^; p^**f**^**
PTSD-RI^g^	31.15 (11.00)	24.69 (12.63)	*t*_(1, 302)_ = 3.51, *p* < 0.001
BDI^h^	21.03 (10.58)	17.21 (11.61)	*t*_(1, 319)_ = 2.31, *p* < 0.001
**SECV scale**^**i**^			
Witnessing	3.66 (2.01)	2.28 (2.23)	*t*_(1, 310)_ = 4.28, *p* < 0.001
Victimization	2.55 (1.66)	1.54 (1.67)	*t*_(1.313)_ = 4.12, *p* < 0.001
**YSR**^**j**^			
Withdrawn	5.55 (2.28)	4.93 (2.73)	*t*_(1, 303)_ = 1.55, p = 0.122
Somatic complaints	5.09 (3.20)	4.02 (3.37)	*t*_(1, 303)_ = 2.15, *p* < 0.05
Anxious/Depressed	11.80 (6.55)	9.51 (6.20)	*t*_(1, 303)_ = 2.45, *p* < 0.05
Social problems	5.51 (2.66)	4.73 (2.60)	*t*_(1, 303)_ = 2.01, *p* < 0.05
Thought problems	4.87 (3.11)	3.96 (3.02)	*t*_(1, 303)_ = 2.01, *p* < 0.05
Attention problems	8.36 (3.19)	6.90 (3.18)	*t*_(1, 303)_ = 3.09, *p* < 0.01
Delinquent behavior	9.02 (3.94)	8.09 (3.77)	*t*_(1, 303)_ = 1.64, *p =* 0.103
Aggressive behavior	15.65 (6.77)	12.50 (6.41)	*t*_(1, 303)_ = 3.27, *p* < 0.001
**TCI**^**k**^			
Novelty seeking	11.53 (2.95)	11.63 (2.97)	*t*_(1, 315)_ = 0.25, ns
Harm avoidance	9.51 (3.16)	8.98 (3.79)	*t*_(1, 315)_ = 0.97, ns
Self-directedness	9.12 (3.66)	10.50 (3.95)	*t*_(1, 315)_ = 2.41, *p* < 0.05
Cooperativeness	13.88 (3.31)	14.32 (3.78)	*t*_(1, 315)_ = 0.82, ns
Self-transcendence	9.47 (3.30)	8.24 (3.31)	*t*_(2, 299)_ = 2.55, *p* < 0.05

a *NSSI, Non-suicidal self-injury*.

b *No–NSSI, No-Non-suicidal self-injury*.

c *M (SD), Mean (Standard Deviation)*.

d *t-test, Student's t-test*.

e *df, Degrees of freedom*.

f *p, Significance value*.

g *PTSD-RI, Post-Traumatic Stress Disorder Reaction Index*.

h *BDI, Beck Depression Inventory*.

i *SECV, Survey of Exposure to Community Violence*.

j *YSR, Youth Self-Report*.

k *TCI, Temperament and Character Inventory*.

## Discussion

This study aimed to determine the prevalence of NSSI among incarcerated adolescents, as well as to explore the potential associations between NSSI and psychopathology and personality. Just over 18% of our subjects reported lifetime engagement in NSSI. The prevalence of NSSI in the current study was lower than the mean prevalence reported in community adolescent samples in international studies where the aggregated lifetime prevalence of NSSI was reported to be as high as 22.1% (13). However, the lifetime prevalence rates in previous studies have varied considerably between countries and in some were as low as 13% (78). In addition, the lifetime prevalence of NSSI in our population was higher than that (13%) reported in the only previous Russian study on the prevalence of NSSI in late adolescents (16). It is uncertain what underlies these differences across studies although Muehlenkamp et al. (10) proposed that dissimilarities in assessment methodologies (including settings and instruments) and in the classification of self-injury might explain the variation in rates between studies.

The number of adolescents with NSSI who reported recurrent thoughts of death and suicidal ideation was over two times higher than those in the No-NSSI group, while almost one-third of the NSSI adolescents also described their previous suicide attempts as serious (i.e., described a definite suicidal intent at the time of the attempt), as compared to only one-tenth of adolescents in the No-NSSI group. These findings demonstrate that while some researchers have suggested that these two types of behavior differ from each other in some fundamental ways (79), they nevertheless often co-occur. This indicates a possible relationship between the engagement of delinquent youths in self-harm and a risk for suicidal behavior. Indeed, these findings are in line with previous reports that suicidal thoughts are more common among self-injurious adolescents, they are more often involved in making suicide attempts (11, 24) and that NSSI may be a predictor of suicidal behavior (80).

Previous research has shown that an abundance of different internalizing and externalizing symptoms are common among individuals with NSSI (36, 81, 82). Findings from our study suggest that incarcerated adolescents with NSSI represent a high-risk group, as they tend to have higher rates of anxiety and PTSD and they also scored higher on almost all types of problem behavior, as assessed by the YSR. In addition, the present study also suggests that community violence exposure is more prevalent among adolescents with NSSI. This further emphasizes the importance of screening for NSSI in juvenile offender populations, especially among those exposed to violence, and that screening protocols should be included in the standardized assessment procedures available in the juvenile justice system.

Although non-significant, the difference in the prevalence of MDD was near significance level, while self-reported depressive symptoms (both the BDI and Anxious/Depressed scale of the YSR) were significantly higher in the NSSI group. This finding is underpinned by earlier studies where depressive symptoms were highly comorbid with NSSI (13, 83). In incarcerated adolescents, MDD symptoms were found among those who reported NSSI twice as often compared to those without a history of self-injury (84). It is not clear why we obtained different results (significant and non-significant) when we assessed the association between NSSI and depression using different measures. However, previous studies that have assessed depressive symptoms by self-reports and clinician-administered interviews have similarly reported varying results. Martin et al. (85) after comparing expert and self-assessments, concluded that self-reports were not reliable in identifying participants with a lifetime diagnosis of MDD. In contrast, although Stuart et al. (86) referred to the clinical interview as the “gold standard” for identifying depression, they also acknowledged that simple self-report methods did have a role when it came to identifying symptoms of depression. Differences in the results between self-reports and clinical interviews might also reflect the sensitive nature of the topic and poor memory due to current mood (87, 88).

The study also examined personality traits in adolescents with NSSI and found, as expected, that they differed from those in No-NSSI adolescents. More specifically, they had lower scores on self-directedness, but higher scores on self-transcendence. Low self-directedness, reflecting unreliability, impulsivity and indecisiveness (76) was recently reported to predict NSSI among adolescents (40), with self-directedness gradually decreasing as the frequency of NSSI increased. As suggested by Glenn and Klonsky (2), most individuals with NSSI have poor impulse control and the engagement of impulsive individuals in NSSI may be related to seeking immediate relief from tension without thinking about the possible consequences of such behavior. There is also some evidence that difficulties in emotion regulation may be associated with low self-directedness (89) and play a role in a number of mental health problems, including NSSI (42). According to Cloninger et al., a person with low self-directedness is characterized by a decreased ability to influence difficult situations and often has problems resolving them positively (45). As a result, when experiencing negative emotions or being in difficult situations, a combination of low self-directedness and emotion regulation difficulties (as suggested by higher anxiety and depression scores), might lead to self-injurious behavior. In connection with this, as Bernheim et al. (90) have previously reported that psychotherapy might play a role in strengthening self-directedness, this might also be important for NSSI treatment, considering that an association has been documented between NSSI and low treatment adherence and low self-directedness (91, 92).

Findings on the association between NSSI and self-transcendence are also in line with those from previous studies where high self-transcendence has been linked to NSSI in adolescents (42, 52, 93). As suggested by Cloninger (76), high self-transcendence is generally associated with extravagant thinking, a lack of logic and unconventional behavior (94, 95) and NSSI might be a result of such traits, where self-transcendence may reflect a sense of detachment between the individual's self and his or her surroundings, potentially leading to self-injurious behavior (93). Previously, it was shown that specific personality traits are not only associated with a predisposition to different mental disorders, but are also able to impact treatment results (96). As low self-directedness and high self-transcendence are common in different psychopathologies, such as personality disorder, depression, bipolar disorder and NSSI (52, 97, 98), then treatment approaches that take these personality traits into account may be relevant.

In spite of important and positive changes in the Russian juvenile justice system in recent years and a dramatic decrease in the number of incarcerated youths (from 18,600 in 2002 to only 1,592 in 2017) (99), it is still unclear whether incarcerated youth receive help which is sufficient to meet their mental health needs. With regard to interventions, evidence-based and developmentally sensitive strategies need to be elaborated to intervene among youth in the juvenile justice system. While evidence from several international studies on the effectiveness of therapeutic interventions is available (100, 101), it is unclear whether these results can be generalized to the Russian context. There is a need therefore for more research in order to address issues related to NSSI among incarcerated youth and gain a better understanding of their current needs and gaps in the provision of services.

Summing up, our findings point to the importance of targeting adolescents in correctional facilities who have engaged in NSSI, as they may represent a high risk group for suicidal behavior. Intervening early to support young people who self-injure, to help prevent an exacerbation of NSSI should be a top priority. In relation to this, our findings may be useful for workers within the juvenile justice system in helping them to identify adolescents with NSSI earlier in order to more quickly initiate intervention measures. Adequate screening and recognition of mental health problems and needs is not only clinically important but may also help to identify factors associated with persistent NSSI and result in more individualized and suitable intervention and prevention efforts, as well as better treatment outcomes.

## Limitations

This study has several limitations which should be mentioned. One of the main limitations and methodological-ethical concerns relates to the data collection setting, where the participants were placed involuntarily. In such an environment it is not clear whether all of the subjects really did think that they had the freedom to refuse to participate and that the information they provided would be guaranteed to be kept confidential. This might have affected their responses. Similarly, in this confined setting the use of self-reports may have resulted in socially desirable responding. Another limitation is related to the lack of information on the diagnostic history of NSSI and that we did not know when the behavior first appeared, before or during the process of incarceration. In addition, we could not determine the directionality of the observed associations given the cross-sectional design of the study. Future longitudinal studies may be helpful both in further elucidating these relationships and also in helping to determine effective prevention and treatment approaches.

## Data Availability Statement

The data used in this study cannot be shared publicly due to the initial decision of the local ethical committee, as well as the restrictions included in the informed consent statement (where it was stated that the data would only be used by the research group and would not be transferred elsewhere). Requests to access the dataset should be directed to Roman Koposov.

## Ethics Statement

The studies involving human participants were reviewed and approved by Northern State Medical University in Arkhangelsk (Russia). Written informed consent to participate in this study was provided by the participants' legal guardian/next of kin.

## Author Contributions

VR, RK, and AS were involved in the conceptualization and design of the study. RK and VR conducted the data collection and drafted the manuscript. VR conducted the data analysis. All authors reviewed and edited the manuscript.

## Conflict of Interest

The authors declare that the research was conducted in the absence of any commercial or financial relationships that could be construed as a potential conflict of interest.
